# Planetary health in general practice: a cross-sectional survey in France

**DOI:** 10.3399/BJGPO.2025.0153

**Published:** 2026-05-06

**Authors:** Charlotte Goolaerts, Eloïse Retailleau, Ludovic Casanova, Gaetan Gentile, Pascal Auquier, Maeva Jego, Thomas Fierling

**Affiliations:** 1 Department of General Practice, Aix-Marseille University, Marseille, France; 2 Center for Studies and Research on Health Services and Quality of Life, Aix-Marseille University, Marseille, France; 3 Institut de Neurosciences des Systèmes, Aix-Marseille University, Marseille, France

**Keywords:** prevention, global health, general practice, environment, primary health care, medical education

## Abstract

**Background:**

In 2019, a global appeal was launched by the World Organization of Family Doctors that invited GPs to take action for planetary health. In 2023, 4 years after this call, what have French GPs done to promote planetary health?

**Aim:**

To describe GPs’ opinions about this subject and pro-planetary health actions in general practice and to evaluate their knowledge of planetary health.

**Design & setting:**

An online survey of GPs, locums, and medical interns in France.

**Method:**

The survey was sent by email to French general practice networks. The responses were statistically analysed, and the demographic data were analysed via descriptive statistics.

**Results:**

The 974 participants who responded placed significant importance on ecology in their personal lives (mean = 7.81/10) and were relatively aware of the environmental impact of health care. In their professional lives, they placed less importance on ecology (mean = 5.80/10). Only 23.10% (*n* = 225) reported being trained on planetary health during their medical education. During consultations regarding both health and environmental benefits, the most frequently given advice involved direct benefits to patients' health. A lack of time (*n* = 676, 69.40%) was the first barrier to integrating planetary health into practice, and the second barrier was a lack of knowledge (*n* = 606, 62.22%).

**Conclusion:**

GPs are sensitive to environmental issues, but they are more detached from these issues in their professional lives. It is crucial to strengthen prevention efforts and integrate environmental considerations into training programmes and health policies to create the conditions necessary for this change in practice.

## How this fits in

In 2019, a global appeal was launched by the World Organization of Family Doctors (WONCA) that invited GPs to take action for planetary health, an evidence-based field of medicine focused on characterising the links between changes in ecosystems owing to human activities and their consequences for health and sustainable approaches to health care. In 2023, 4 years after this call, this study showed that French GPs are citizens who are sensitive to environmental issues; however, they are also health professionals who are more detached from these issues and have difficulty translating them into concrete action. To reconcile citizens and healthcare professionals, it seems necessary to offer medical training on planetary health and to involve global public health policies.

## Introduction

Climate change threatens the health of humanity^1^ as well as the progress in health and development achieved over the past 50 years.^2^

In recent decades, several concepts have emerged that link the environment and health. Environmental health,^3^ first defined in the late 20th century, focuses on how environmental factors affect human disease. ‘One health’ emphasised the interconnection between human, animal, and environmental health. In 2015, planetary health^4,5^ went further, stressing that the wellbeing of all life depends on the stability of Earth’s life-support systems.

As frontline players in the healthcare system, GPs know their patients’ family, economic, social, professional, and natural environments. GPs’ role as primary care providers and referral agents, combined with the holistic, person-centred approach at the heart of their skills, make them key players in somatic prevention at the psychological, social, and environmental levels.^6^ In fact, GPs play a central role in promoting planetary health initiatives with health co-benefits.^7,8^

In 2019, WONCA, in association with the Planetary Health Alliance, issued the first call urging GPs worldwide to act for planetary health.^9^ They encouraged GPs to combat the acceleration of climate change by practising sustainable health care, strengthening prevention, raising patient and community awareness about planetary health, and preparing responses to the negative consequences of the climate crisis.^10–12^ Since then, this field has received increasing interest among French practitioners. In 2023, where did we stand 4 years after this call to action? In this context, it seemed important to study the care practices of GPs in relation to planetary health.

The primary objective of this study was to describe the actions supporting planetary health that were practised by French GPs in 2023. The secondary objective was to assess knowledge of suggested actions and the challenges faced by GPs in implementing them.

## Method

### Study design

This was a cross-sectional questionnaire-based study delivered via the online platform https://framaforms.org from 21 June 2023 to 2 October 2023. It was distributed by email through several organisations: Departmental Councils of the Medical Order, Territorial Multi-Professional Health Communities, Regional Health Agencies, Regional Unions of Private Practice Physicians, and various universities. The participants were asked to disclose their demographic information, their perceptions of the role that ecology plays in their personal and professional lives, their opinions on planetary health and their eco-responsibility, and the barriers they perceived in integrating planetary health into their practices.

### Study population

The inclusion criterion was practising as an established GP or as a locum in France. In addition to established GPs, medical interns with at least one outpatient rotation were included. Their participation provided the perspective of future healthcare professionals, who are often more attuned to environmental issues because of training in global health. This comparative approach helps to identify generational shifts and anticipate future trends in general practice.

The exclusion criteria were not being a GP, practising outside France, or working in a hospital setting. General medicine interns practising abroad or who had not completed an outpatient rotation were also excluded.

A target sample size of 710 was calculated.

### Data collection

The questionnaire was based on a literature review and consisted of 25–28 questions, depending on the participant’s professional status.

Consent was requested in the first question, which was mandatory to access the survey. The questions were closed-ended, with either single- or multiple-choice options. A four-point Likert scale was used for most items, and a 0–10 scale was used for certain items to precisely study the opinions of the physicians. The questions were divided into five different sections that examined the role of ecology in the personal and professional lives of the physicians, their opinions on the role of GPs in planetary health, the frequency of practices with co-benefits, eco-responsibility in practice, and sociodemographic characteristics. To explore the GPs’ opinions on the limits of their role, the planetary health professional recommendations issued by WONCA and the College of General Medicine France were presented to participants for evaluation.^5,13^

### Statistical analysis

Descriptive statistics were used to examine the distribution of responses, expressed as numbers and percentages, among participants in the investigated categories (‘strongly agree’, ‘agree’, ‘disagree’, and ‘strongly disagree’) in relation to the different questions.

*P*-values were used to assess differences in practice or opinions between participants, and were calculated via the χ^2^ test.

After the study population was subdivided according to professional status (that is, intern, locum, and practising physician), Student’s *t*-tests, Pearson correlation tests, and variance analyses were performed with the alpha error risk set at 5%.

All analyses were conducted using R Studio (version 4.2.2). The study was reported according to the Strengthening the Reporting of Observational Studies in Epidemiology guidelines.

## Results

### Population characteristics

A total of 974 participants (66.12% female and 33.88% male) completed the questionnaire (Table 1). The age of participants ranged from 24 to 84 years, with an average age of 41.34 years. The 32–50-year age group accounted for 50% of the total sample. A majority (64.37%) of the participants had ≥1 child (*n* = 627).

**Table 1.** Participant characteristics (*N* = 974)

Characteristic		*n* (%)	Mean	Median	SD	Min–max
Sex	Male	330 (33.88)				
	Female	644 (66.12)				
Age, years			41.34	38	12.7	24–84
Have child	Yes	627 (64.37)				
	No	347 (35.63)				
Professional status	Established GP	708 (72.69)				
	Locum GP	96 (9.86)				
	Medicine interns	170 (17.45)				
Exercise period, year			14.88	11	11.7	0–60
Consultation time, min			19.06	20	4.47	10–40

Max = maximum. Min = minimum. SD = standard deviation.

### Knowledge of health‒environment links

Knowledge of the field of planetary health (Table 2) was divided, with 49.28% lacking knowledge of this field. Most participants (74.85%) were not familiar with the ‘one health’ concept. In contrast, most doctors (78.13%) were familiar with the concept of environmental health. There was no significant difference in knowledge of the three concepts according to the participants' professional status (analysis of variance, *P* = 0.65) or sex (Student’s *t*-test, *P* = 0.82).

**Table 2.** Participants’ knowledge of the concepts and training

Concept	**Yes, *n* (%)**		**No, *n* (%)**
**One health**			
Established GP	169 (23.87)		539 (76.13)
Locum GP	30 (31.25)		66 (68.75)
Medicine interns	46 (27.06)		124 (72.94)
Total	245 (25.15)		729 (74.85)
**Environmental health**			
Established GP	566 (79.94)		142 (20.06)
Locum GP	73 (76.04)		23 (23.96)
Medicine interns	122 (71.76)		48 (28.24)
Total	761 (78.13)		213 (21.87)
**Planetary health**			
Established GP	337 (47.60)		371 (52.40)
Locum GP	52 (54.17)		44 (45.83)
Medicine interns	91 (53.53)		79 (46.47)
Total	480 (49.28)		494 (50.72)
**Level of training**			
	**Initial training**	**Continuous training**	**No training**
Established GP	30 (4.24)	131 (18.50)	547 (77.26)
Locum GP	10 (10.42)	17 (17.71)	69 (71.88)
Medicine interns	31 (18.24)	6 (3.53)	133 (78.24)
Total	71 (7.29)	154 (15.81)	749 (76.90)

Only 23.10% (*n* = 225) of the participants reported being trained in the links between health and the environment, and most reported being trained during their continuing medical education (15.81%, *n* = 154) (Table 2). 

A total of 47.64% (*n* = 464) of participants were able to correctly identify greenhouse gas emissions. Only 8.52% of the surveyed physicians were familiar with the PBT (persistent, bioaccumulative, and toxic substances) index, which classifies the impact of drugs on the aquatic environment according to three criteria: persistence, bioaccumulation, and toxicity (data not shown).^14^

### Importance given to the environment

Most of the surveyed physicians expressed interest in the subject of this study. Locums were more interested in the subject than established physicians. Females (Table 3) and participants without children (data not shown) reported being more interested in the topic of planetary health.

**Table 3.** Importance given to planetary health by participants

Importance given to planetary health in participant’s personal lives	Mean	Median	SD	*P*-value
By professional status				
Established GP	7.82	8	1.64	0.37
Locum GP	7.95	8	1.52	
Medicine intern	7.67	8	1.63	
By sex				
Male	7.53	8	3.33	0.0002^a^
Female	7.95	8	2.23	
Total	7.81	8	2.64	—
**Importance given to planetary health in participant’s professional lives**				
By professional status				
Established GP	5.93	6	2.24	0.001^a^
Locum GP	5.65	6	2.23	
Medicine intern	5.27	5	2.08	
By sex				
Male	5.62	6	2.44	0.09
Female	5.88	6	2.10	
Total	5.80	6	2.22	—

^a^ *P*<0.001. SD = standard deviation.

The participants, especially females (*P* = 0.0002), reported that ecology played an important role in their personal lives, with no difference in professional status (*P* = 0.37). The surveyed physicians reported generally applying planetary health-related prevention advice in their own lives (Table 3).

In their professional daily lives, the importance placed on ecology was lower, with no difference in terms of sex. The level of adherence to advice that was beneficial to their health and the environment differed significantly according to the professional status of the participants, with medical interns applying this advice less frequently in their professional lives (*P* = 0.001) (Table 3).

### The role of the GP in consultations

The participants mostly agreed that raising patient awareness (Table 4) about the impacts of improper medication use (*n* = 767, 78.75%), providing advice for healthier, more sustainable diets (*n* = 621, 63.76%) and identifying exposure to environmental risk in their patients' personal or professional lives (*n* = 567, 58.21%) were part of their role as GPs. The surveyed physicians mostly agreed with regard to their role in providing information on the health effects of pollution and climate change (*n* = 813, 83.47% agreed or somewhat agreed). However, they were ambivalent about raising patient awareness of the health and environmental impacts of major modes of transportation (*n* = 496, 50.92% agreed or somewhat agreed).

**Table 4.** The role of the GP in consultations

The role of the GP in consultations is:	Disagree, *n* (%)	Somewhat disagree, *n* (%)	Somewhat agree, *n* (%)	Agree, *n* (%)
To recommend a more sustainable and healthy diet	9 (0.92)	34 (3.49)	310 (31.83)	621 (63.76)
To identify personal and professional environmental risk exposures	16 (1.64)	63 (6.47)	328 (33.68)	567 (58.21)
To raise awareness about endocrine disruptors	13 (1.33)	54 (5.54)	412 (42.30)	495 (50.82)
To raise awareness about the impacts of improper medication use	4 (0.41)	12 (1.23)	191 (19.61)	767 (78.75)
To provide information on the health effects of pollution and climate change	39 (4.00)	122 (12.53)	433 (44.46)	380 (39.01)
To raise awareness of the health and environmental impacts of modes of transport	188 (19.30)	290 (29.77)	341 (35.01)	155 (15.91)
To advise patients to buy eco-responsible or eco-labelled products	286 (29.36)	387 (39.73)	231 (23.72)	70 (7.19)
To advise patients to reduce their environmental impact	231 (23.72)	402 (41.27)	252 (25.87)	89 (9.14)

### Planetary health-related prevention practices in consultation

The subject of the interconnections among human activities, health, and the environment was addressed daily during consultations by 12.53% (*n* = 122) of the participants. The frequency with which this topic was discussed did not vary significantly according to the length of the consultation (data not shown).

With regard to alimentation, the advice most frequently mentioned by the GPs concerned avoidance of processed products. Avoiding the consumption of processed food products such as refined cereals, which contain additives and/or added sugars, was advised daily by 63.96% (*n* = 623) of the participants. Limiting animal proteins was advised at least once a week by 34.60% of the doctors (*n* = 337) and daily by 32.55% (*n* = 317). Prevention of the harmful effects of contact between food and plastic was never addressed by 41.68% (*n* = 406) of the doctors surveyed. Similarly, 48.87% (*n* = 476) of the participating doctors said that they never discussed food waste reduction with their patients (Table 5).

**Table 5.** Advice discussed during consultations with patients

Advice category	Never, *n* (%)	Rarely (<1 x/week), *n* (%)	Sometimes (≥1 x/week), *n* (%)	Often (≥1 x/day), *n* (%)
**In promoting healthy and sustainable eating**				
Avoid processed food products	38 (3.90)	65 (6.67)	248 (25.46)	623 (63.96)
Limit animal proteins	134 (13.76)	186 (19.10)	337 (34.60)	317 (32.55)
Buy seasonal foods	136 (13.96)	184 (18.89)	317 (32.55)	337 (34.60)
Avoid contact between food and plastic	406 (41.68)	288 (29.57)	191 (19.61)	89 (9.14)
Reduce food waste	476 (48.87)	278 (28.54)	137 (14.07)	83 (8.52)
Prefer local products	239 (24.54)	238 (24.44)	278 (28.54)	219 (22.48)
Prefer organic products	283 (29.06)	254 (26.08)	294 (30.18)	143 (14.68)
Drink tap water	239 (24.54)	220 (22.59)	249 (25.56)	266 (27.31)
**To promote perinatal health**				
Do not perform home improvement during pregnancy	336 (34.50)	280 (28.75)	218 (22.38)	140 (14.37)
Avoid smoking indoors or close to children or pregnant women	7 (0.72)	32 (3.29)	122 (12.53)	813 (83.47)
Limit the amount of household products	354 (36.34)	261 (26.80)	212 (21.77)	147 (15.09)
Do not heat food or liquids in plastic vessels	307 (31.52)	260 (26.69)	233 (23.92)	174 (17.86)
Open the windows twice a day to reduce indoor air pollution	204 (20.94)	232 (23.82)	295 (30.29)	243 (24.95)
Avoid the use of insecticides and air fresheners	237 (24.33)	251 (25.77)	235 (24.13)	251 (25.77)
Limit the number of hygiene products used	218 (22.38)	231 (23.72)	243 (24.95)	282 (28.95)
Pregnant women should avoid essential oils, colorants, and nail polish	220 (22.59)	214 (21.97)	237 (24.33)	303 (31.11)
**Impact of medicines on health and the environment**				
Medication side effects	2 (0.21)	13 (1.33)	166 (17.04)	793 (81.42)
Possible drug interactions	3 (0.31)	43 (4.41)	282 (28.95)	646 (66.32)
Risk of antibiotic resistance	4 (0.41)	28 (2.87)	203 (20.84)	739 (75.87)
The impact of drugs on biodiversity	477 (48.97)	276 (28.34)	164 (16.84)	57 (5.85)
Water and soil pollution by drugs	417 (42.81)	288 (29.57)	192 (19.71)	77 (7.91)
The need to return unused medicines to the pharmacy	86 (8.83)	226 (23.20)	362 (37.17)	300 (30.80)
Explain the natural evolution of diseases	3 (0.31)	30 (3.08)	153 (15.71)	788 (80.90)
Offer initial non-medication management whenever possible	4 (0.41)	27 (2.77)	181 (18.58)	762 (78.23)
Choosing narrow-spectrum antibiotics	3 (0.31)	4 (0.41)	56 (5.75)	911 (93.53)
Reassessing treatments and deprescribing prescriptions	1 (0.10)	25 (2.57)	251 (25.77)	697 (71.56)
Prefer dry powder inhalers over aerosol inhalers	320 (32.85)	226 (23.20)	214 (21.97)	214 (21.97)
**Other advice**				
Recycle	593 (60.88)	214 (21.97)	133 (13.66)	34 (3.49)
Compost	620 (63.66)	211 (21.66)	117 (12.01)	26 (2.67)
Prefer public transport	524 (53.80)	253 (25.98)	158 (16.22)	39 (4.00)
Avoid fossil fuels for heating and cooking	723 (74.23)	185 (18.99)	55 (5.65)	11 (1.13)
Perform an energy audit of the home	761 (78.13)	177 (18.17)	32 (3.29)	4 (0.41)

With respect to perinatal care, avoiding smoking indoors or close to children or pregnant women was the prevention message most frequently mentioned by participants (daily in consultation by 83.47%*, n* = 813). The advice to avoid heating food or liquid in plastic vessels was never recommended by 31.52% (*n* = 307) of the participants (Table 5).

With respect to drug prescriptions, the majority of the doctors said that they discussed treatment side effects (81.42%, *n* = 793), drug interactions (66.32%, *n* = 646), and antibiotic resistance (75.87%, *n* = 739) with their patients daily. The impact of medicines on biodiversity was discussed rarely (48.97% never, *n* = 477), as was the pollution of water and soil by medicines (42.81% never, *n* = 417) (Table 5).

Soft mobility was the most frequently recommended piece of advice (at least once a week by 23.92% [*n* = 233]) of the participating GPs and at least once a day by 68.79% (*n* = 670). Overall, 37.17% (*n* = 362) of the participants said they ‘prescribed’ nature to their patients at least once a day (data not shown).

### Attenuation actions

Five actions were presented to the participating doctors: recycling, composting, using public transport, performing a home energy diagnosis, and limiting the use of fossil fuels for heating or cooking. The majority of the GPs surveyed said that they never gave their patients this advice during consultation (Table 5).

### Eco-responsibility

With respect to waste management practices, a majority (71.61%, *n* = 507) of the established GPs said that they sorted their waste every day (data not shown).

In terms of business travel, a majority of the established GPs (61.72%, *n* = 437) said that they systematically grouped their home visits, and approximately one-third said that they systematically made journeys of less than one kilometre on foot or by bicycle (data not shown).

The majority of doctors discussed the natural course of disease with their patients daily (80.90%, *n* = 788), proposed initial non-drug management whenever possible (78.23%, *n* = 762, and prescribed narrow-spectrum antibiotics (93.53%, *n* = 911). Reassessment of prescribed treatments and deprescribing were conducted daily by 71.56% (*n* = 697) of the doctors surveyed. Dry powder inhalers were prescribed instead of aerosols by only 21.97% of the participants daily or at least once a week, respectively (Table 5).

### Main obstacles

The GPs perceived the main obstacles to integrating planetary health into their consultations to be a lack of time (69.40%, *n* = 676), a lack of knowledge (62.22%, *n* = 606), and a feeling of powerlessness in the face of climate-related issues (37.17%, *n* = 362) (Table 6).

**Table 6.** Main obstacles

Obstacle	Medicine intern (*N* = 170), *n* (%)	Locum GP (*N* = 96), *n* (%)	Established GP (*N* = 708), *n* (%)	Total (*N* = 974), *n* (%)
Lack of time	107 (62.94)	75 (78.13)	494 (69.77)	676 (69.40)
Lack of knowledge	131 (77.06)	59 (61.46)	416 (58.76)	606 (62.22)
Feeling of powerlessness	82 (48.24)	43 (44.79)	237 (33.47)	362 (37.17)
Not the role of GPs	58 (34.12)	25 (26.04)	242 (34.18)	325 (33.37)
Difficulty changing habits	55 (32.35)	25 (26.04)	208 (29.38)	288 (29.57)
Financial aspects	46 (27.06)	30 (31.25)	199 (28.11)	275 (28.23)
Politicised subject	37 (21.76)	19 (19.79)	147 (20.76)	203 (20.84)
Lack of interest	20 (11.76)	2 (2.08)	45 (6.36)	67 (6.88)

## Discussion

### Summary

Our study revealed a significant gap between French GPs’ strong personal interest in environmental issues and the integration of planetary health principles into their professional practice. Actions taken by GPs in the field of planetary health are often ad hoc rather than systematic. They focus primarily on good clinical practices that offer health benefits for their patients rather than directly reducing their environmental impact.

The participants identified several major obstacles, including a lack of time to address planetary health during consultations, insufficient training, and a sense of powerlessness. However, the frequency with which planetary health was discussed did not vary significantly with the length of consultations. One of the main reasons cited by GPs for their reluctance to engage in planetary health was the perception that it is a politicised and potentially polarising topic.

### Strengths and limitations

This study addresses a topical issue for which few studies exist. Sources of information consist primarily of dissertations, family medicine theses, guides, and websites.

This is the first quantitative study in France to describe GPs’ actions in support of planetary health in the care of their patients on a large scale. This study may form an initial basis for national practices in France.

However, this study has several limitations. The response rate was difficult to determine because of the diversity of distribution channels. Furthermore, this study presents biases with regard to recruitment and social desirability.

### Comparison with existing literature

The participants in our study expressed interest in planetary health. On the whole, GPs are concerned about their role in planetary health.

As observed in the literature, females in our study showed significantly greater interest in the subject and were more likely to integrate ecological principles into their daily lives than males.^15–17^

In France, individuals aged 40–49 years appear to be the most concerned about protecting the environment for future generations.^18^ This may explain why our study, like previous studies,^17,19^ included a sample population younger than the target demographic. However, no correlation was found between the participants' age and their initial level of interest in the subject in our study.

There was a significant discrepancy between the role of ecology in the personal and professional lives of our participants, a finding that was also reported in the literature. Although medical interns place significantly less emphasis on ecology in their practice, they are equally convinced of their role in planetary health.^17,20^

Few large-scale studies have described the actions recommended for planetary health by French GPs. Our results are comparable with those of the few previous studies.^21,22^

### Implications for research and practice

Although GPs are concerned about climate change, they report a feeling of powerlessness in the face of environmental challenges^23^ and a lack of time to discuss this topic during consultations.

A lack of knowledge, which is inherent in a lack of training, is a major obstacle to the involvement of GPs.^17,20,23–26^ Earlier studies recommended improving training at several levels, both for established doctors and for medical students, by offering continuing education.^27^ Studies have stressed the importance of preparing future generations of healthcare professionals to better understand planetary health issues and the health consequences of exceeding planetary limits. Furthermore, emphasis has been placed on identifying the most vulnerable populations in the face of ongoing climate change.^11,28^

Moreover, the literature highlights the need for GPs to set an example for both their patients and their colleagues.^22,29^ As practice managers and leaders, they are responsible for raising awareness among other healthcare professionals and making decisions that support planetary health within their practice.^20,22^ While GPs recognise the importance of sustainable development in society, implementing lasting change at an individual level remains challenging. To facilitate this transition, they require tools and support, particularly from authorities.^23^

GPs are citizens who are sensitive to environmental issues. At the same time, they are healthcare professionals who are more detached from these issues and have difficulty translating them into concrete actions. To reconcile citizens and healthcare professionals, it seems necessary to offer medical training on planetary health and to involve more global public health policies. A study with mixed methods of the factors that favour or limit the integration of planetary health actions by GPs, and patients' acceptance of these measures, would make it possible to offer support in changing GPs’ practices and patients’ acceptance.

## Data

The dataset relied on in this article is available from the corresponding author on reasonable request.
